# Illness-related stigma mediates the association between disease severity and dermatology- specific quality of life in chronic inflammatory skin diseases: a structural equation modeling study

**DOI:** 10.3389/fmed.2026.1821716

**Published:** 2026-05-07

**Authors:** Xiaoya Fang, Xuan Zhou, Dayan Meng, Xiaodan Zhao, Xiaonan Zhou, Yan Zong

**Affiliations:** 1 Department of Nursing, Hospital for Skin Diseases, Institute of Dermatology, Chinese Academy of Medical Sciences and Peking Union Medical College, Nanjing, China; 2 Department of Dermatologic Surgery, Hospital for Skin Diseases, Institute of Dermatology, Chinese Academy of Medical Sciences and Peking Union Medical College, Nanjing, China; 3 Department of Dermatology Ward II, Hospital for Skin Diseases, Institute of Dermatology, Chinese Academy of Medical Sciences and Peking Union Medical College, Nanjing, China

**Keywords:** chronic inflammatory skin diseases, health-related quality of life, hope, illness-related stigma, perceived social support, structural equation modeling

## Abstract

**Background:**

Chronic inflammatory skin diseases substantially impair health-related quality of life (HRQoL). However, clinical severity alone does not fully account for variability in patient-reported outcomes.

**Objectives:**

To test a theory-driven pathway model linking disease severity to HRQoL via illness-related stigma and to examine the roles of hope and perceived social support.

**Methods:**

In this single-center cross-sectional study (*n* = 404), disease severity was assessed using affected body surface area (BSA), itch and skin pain. Illness-related stigma (8-item Stigma Scale for Chronic Illnesses, SSCI-8), hope (Herth Hope Index, HHI), perceived social support (Multidimensional Scale of Perceived Social Support, MSPSS) and HRQoL (Dermatology Life Quality Index, DLQI) were assessed using validated Chinese-language versions. Structural equation modeling with maximum likelihood estimation and bias-corrected bootstrapping (5,000 resamples) was performed.

**Results:**

The model demonstrated good fit (χ^2^/df = 2.04; CFI = 0.977; TLI = 0.969; RMSEA = 0.051; SRMR = 0.058). Greater BSA (β = 0.30), itch (β = 0.20) and pain (β = 0.20) were associated with higher stigma (all *P* < 0.001). Stigma was associated with lower hope (β = −0.53) and worse dermatology-specific HRQoL (higher DLQI scores; β = 0.44) (both *P* < 0.001). Hope (β = −0.30, *P* < 0.001) and perceived social support (β = −0.13, *P* = 0.007) were independently associated with better dermatology-specific HRQoL. The model accounted for 16.1% of the variance in stigma, 28.0% in hope, and 44.0% in dermatology-specific HRQoL.

**Conclusion:**

Disease burden may influence HRQoL partly through illness-related stigma and reduced hope. These findings support integrating psychosocial assessment into routine dermatologic care.

## 1 Introduction

Chronic inflammatory skin diseases comprise a heterogeneous group of persistent, immune-mediated dermatoses characterized by relapsing or chronic courses, sustained inflammatory activity and visible lesions that substantially impair daily functioning and psychosocial well-being. Prominent examples include psoriasis, atopic dermatitis and other eczematous dermatoses, chronic urticaria and autoimmune bullous diseases, which are marked by immune dysregulation, symptom burden (e.g., itch and pain) and recurrent flares. Collectively, skin and subcutaneous diseases are a leading cause of non-fatal health loss globally and contribute substantially to healthcare utilization (1, 2).

In clinical practice, disease severity is commonly quantified using objective measures such as affected body surface area (BSA) and patient-reported symptoms, including itch and pain. However, disease severity explains only a proportion of the variance in health-related quality of life (HRQoL). Patients with comparable objective severity frequently report markedly different HRQoL outcomes, suggesting that psychosocial processes play a critical role in shaping patient experience (3, 4). Within a biopsychosocial framework, clinical severity reflects biological disease activity, whereas psychological and social factors may influence how disease translates into lived burden.

Stigma is a particularly salient psychosocial construct in visible skin disease. It encompasses both enacted or anticipated social rejection and internalized negative self-evaluations that influence coping, identity and social functioning. Multinational studies have documented high levels of perceived stigmatization across diverse dermatological conditions, with greater clinician-assessed severity and symptom burden associated with increased stigma (5). These findings are consistent with conceptual models of stigma, in which disease-related social stressors and internalized devaluation may influence quality of life through cognitive and emotional pathways, suggesting that stigma may function as a mediator linking disease severity to HRQoL (6, 7). In addition, the extent to which stigma translates into poorer HRQoL may depend on the availability of psychological and interpersonal resources that support adaptation to chronic disease. Such resources may help explain why patients with similar levels of clinical severity experience different levels of impairment in dermatology-specific HRQoL.

Psychological and interpersonal resources may buffer these adverse effects. Hope, conceptualized as a future-oriented cognitive–emotional resource characterized by goal-directed thinking and positive expectations, has been identified as an important adaptive resource in chronic illness populations and is associated with better psychological adjustment and quality of life (8). Perceived social support, defined as the subjective sense of being cared for and supported by family, friends or significant others, is similarly linked to reduced psychological distress and improved well-being among individuals with chronic health conditions, including dermatological disorders (9). Hope may therefore represent an internal psychological resource, whereas perceived social support may operate as a broader interpersonal context that buffers stress and facilitates adjustment. Nevertheless, integrative models examining how disease severity, stigma and psychosocial resources jointly influence dermatology-specific HRQoL remain scarce, particularly within dermatology populations.

Structural equation modeling (SEM) provides a theory-driven and flexible approach to simultaneously test interconnected pathways and indirect effects (10). Building on empirical findings and a biopsychosocial perspective, we hypothesized that illness-related stigma would mediate the association between disease severity (objective and symptom-based indicators) and dermatology-specific HRQoL, and that hope and perceived social support would exert independent and potentially indirect effects on HRQoL. The present study therefore tested a theory-driven pathway model linking BSA, itch and pain to HRQoL via illness-related stigma, while examining the roles of hope and perceived social support.

## 2 Materials and methods

### 2.1 Study design, participants, and procedure

This single-center, cross-sectional study was conducted at the Hospital for Skin Diseases, Institute of Dermatology, Chinese Academy of Medical Sciences & Peking Union Medical College (Nanjing, China). Consecutive inpatients with chronic inflammatory skin diseases were recruited between August 2024 and April 2025.

Eligibility criteria were: (i) a confirmed diagnosis of chronic inflammatory skin disease by dermatologists; (ii) age ≥ 18 years; and (iii) ability to understand and complete the questionnaires independently. Patients with severe cognitive impairment or major psychiatric disorders that could compromise informed consent or questionnaire validity were excluded.

The study protocol was approved by the Ethics Committee of the Hospital for Skin Diseases, Institute of Dermatology, Chinese Academy of Medical Sciences and Peking Union Medical College (approval no. 2024-055). All participants provided written informed consent prior to enrolment. A total of 404 patients were included in the final analysis. All questionnaires were completed in paper-based format during hospitalization. Questionnaire data were independently double-entered by two researchers to ensure accuracy.

In addition to the study variables specified in the structural equation model, demographic and clinical background characteristics were collected to describe the study sample, including sex, age, disease category, educational level, marital status, self-reported financial burden, smoking status, alcohol consumption, comorbid medical history, body mass index (BMI), and disease duration. Financial burden was self-rated as low, moderate, high, or very high. Smoking status and alcohol consumption were classified as current, never, or former. Comorbid medical history was self-reported and recorded as yes or no, referring to the presence of previously diagnosed medical conditions in addition to the current dermatological disease. Lesion locations were also recorded. In an exploratory supplementary analysis, lesion location was additionally grouped into visible site involvement (head, face, neck, or hands) and high-impact site involvement (perineal, intertriginous, mucosal, or foot sites).

### 2.2 Measures

All instruments were administered in validated Chinese versions.

#### 2.2 1 Disease severity indicators

Objective disease severity was assessed using affected body surface area (BSA, %), evaluated by board-certified dermatologists. Subjective symptom severity was assessed using 11-point numerical rating scales (NRS; 0 = no symptom, 10 = worst imaginable) for itch and skin pain, with higher scores indicating greater intensity. These three indicators were treated as exogenous observed predictors in the structural equation model.

#### 2.2.2 Illness-related stigma

Illness-related stigma was assessed using the 8-item Stigma Scale for Chronic Illnesses (SSCI-8) (11). The Chinese version of the SSCI has demonstrated acceptable psychometric properties in Chinese clinical populations (12). The total score was specified as an observed variable in the SEM. Higher scores indicate greater perceived stigma. Internal consistency in the present sample was excellent (Cronbach’s α = 0.951).

#### 2.2.3 Hope

Hope was measured using the Herth Hope Index (HHI) (13). The Chinese-language version has demonstrated acceptable reliability and validity in Chinese populations (14). In the structural model, hope was specified as a latent construct indicated by its three established dimensions: Temporality and Future, Positive Readiness and Expectancy, and Interconnectedness. Higher scores indicate greater hope. Internal consistency in the present sample was good (Cronbach’s α = 0.896).

#### 2.2.4 Perceived social support

Perceived social support was assessed using the Multidimensional Scale of Perceived Social Support (MSPSS) (15). The validated Chinese version has demonstrated good psychometric properties in Chinese samples (16). In the SEM, perceived social support was specified as a latent construct indicated by its three subscales (Family, Friends, Significant Others). Higher scores reflect greater perceived social support. Internal consistency was excellent (Cronbach’s α = 0.936).

#### 2.2.5 Dermatology-specific HRQoL

Dermatology-specific health-related quality of life was assessed using the Dermatology Life Quality Index (DLQI) (17). The Chinese-language version has demonstrated acceptable psychometric properties in dermatology samples (18). The total score (range: 0–30) was specified as an observed outcome variable in the SEM. Higher scores indicate greater impairment. Internal consistency in the present sample was excellent (Cronbach’s α = 0.906).

### 2.3 Statistical analysis

Descriptive statistics were calculated for demographic and clinical characteristics (Table 1) and for the study variables included in the structural equation model (Table 2). To provide a fuller description of the study variables, both mean ± standard deviation and median (interquartile range) are reported. Distributional characteristics were examined using skewness and kurtosis statistics. Given the ordinal nature of several measures and evidence of non-normal distributions, Spearman’s rank-order correlation coefficients were used to examine bivariate associations among study variables (Table 3).

**TABLE 1:** Demographic and clinical characteristics of the study participants (*N* = 404).

Characteristic	Category	Value
Gender, n (%)	Male	258 (63.9)
	Female	146 (36.1)
Age, years; median (IQR)		61 (46–73)
Disease type, n (%)	Eczema	82 (20.3)
	Psoriasis	110 (27.2)
	Atopic dermatitis	71 (17.6)
	Autoimmune bullous disease	67 (16.6)
	Photosensitive dermatosis	43 (10.6)
	Chronic urticaria	31 (7.7)
Education level, n (%)	Primary school or below	114 (28.2)
	Junior high school	129 (31.9)
	Senior high school/vocational	85 (21.0)
	Junior college	41 (10.2)
	Undergraduate	30 (7.4)
	Postgraduate or above	5 (1.2)
Marital status, n (%)	Unmarried	45 (11.1)
	Married	348 (86.1)
	Divorced/widowed	11 (2.7)
Financial burden, n (%)	Low	190 (47.0)
	Moderate	142 (35.1)
	High	68 (16.8)
	Very high	4 (1.0)
Smoking status, n (%)	Current	102 (25.2)
	Never	264 (65.3)
	Former	38 (9.4)
Alcohol consumption, n (%)	Current	30 (7.4)
	Never	330 (81.7)
	Former	44 (10.9)
Comorbid medical history, n (%)	Yes	274 (67.8)
	No	130 (32.2)
BMI, kg/m^2^; median (IQR)		23.8 (21.6–26.1)
Disease duration, months; median (IQR)		12 (2–60)

Values are presented as n (%) unless otherwise indicated. Percentages may not sum to 100% due to rounding. BMI, body mass index; IQR, interquartile range.

**TABLE 2:** Descriptive statistics of the study variables (*N* = 404).

Variable	Mean ± SD	Median (IQR)	Skewness	Kurtosis
Affected BSA (%)	35.54 ± 23.73	32.00 (37.00)	0.54	−0.49
Itch severity	4.23 ± 1.65	5.00 (3.00)	−0.47	−0.73
Skin pain severity	1.39 ± 1.70	0.00 (2.00)	1.07	0.38
Illness-related stigma	16.47 ± 8.37	14.00 (13.00)	0.91	−0.14
Hope	35.80 ± 6.40	36.00 (6.00)	−1.47	3.49
Perceived social support	30.90 ± 12.50	29.00 (15.00)	0.77	0.49
DLQI score	18.36 ± 7.05	19.50 (12.00)	−0.40	−0.80

Values are presented as mean ± standard deviation or median (interquartile range). Skewness and kurtosis are reported to describe distributional characteristics. BSA, body surface area; DLQI, Dermatology Life Quality Index; IQR, interquartile range.

**TABLE 3:** Spearman’s rank-order correlation coefficients among study variables (*N* = 404).

Variable	1	2	3	4	5	6	7
1. Affected BSA (%)	1	1	1	1	1	1	1
2. Itch severity	0.08						
3. Skin pain severity	−0.02	−0.08					
4. Illness-related stigma	0.28**	0.28**	0.11*				
5. Hope	−0.04	−0.17**	−0.05	−0.39**			
6. Perceived social support	0.11*	0.02	0.06	−0.01	−0.05		
7. DLQI score	0.12*	0.20**	0.07	0.64**	−0.46**	−0.07	

Values are Spearman’s rank-order correlation coefficients (two-tailed). *P < 0.05; **P < 0.01.

SEM was conducted using IBM SPSS AMOS (version 28.0; IBM Corp., Armonk, NY, United States) with maximum likelihood estimation. Given the large sample size, maximum likelihood estimation was considered robust to moderate deviations from normality. In the final model, affected body surface area (BSA, %), itch severity and skin pain severity were specified as exogenous observed variables. Illness-related stigma (SSCI-8 total score) and DLQI total score were specified as manifest observed variables. Hope and perceived social support were specified as latent constructs, indicated by their respective subscales, as detailed in Supplementary Table S1.

A theory-driven baseline model was specified according to the hypothesized relationships among disease severity indicators, illness-related stigma, psychological resources (hope and perceived social support), and dermatology-specific quality of life. The prespecified baseline model is shown in Supplementary Figure S1. Alternative specifications were evaluated for theoretical plausibility and statistical support. Direct paths from severity indicators to DLQI and from perceived social support to hope were tested but were not statistically significant and were therefore removed for parsimony. Covariances among exogenous severity indicators were freely estimated in the initial model; only the residual covariance between itch severity and skin pain severity was statistically significant and retained, whereas other non-significant covariances were constrained to zero. Nested model comparisons indicated that these refinements did not meaningfully impair model fit (ΔCFI < 0.01).

Inference for structural paths and mediation effects was based on bias-corrected (BC) bootstrapping with 5,000 resamples. Two-tailed BC P values and BC 95% confidence intervals (CIs) are reported for direct and indirect effects (Tables 4, 5). Indirect effects were considered statistically significant when the BC 95% CI did not include zero. Statistical significance was defined as a two-tailed α of 0.05.

**TABLE 4:** Direct effects in the final structural equation model based on bias-corrected bootstrapping.

Path	Standardized β	BC 95% CI	Two-tailed P
Affected BSA (%) → Illness-related stigma	0.30	0.21 to 0.39	< 0.001
Itch severity → Illness-related stigma	0.20	0.11 to 0.29	< 0.001
Skin pain severity → Illness-related stigma	0.20	0.10 to 0.31	< 0.001
Illness-related stigma → Hope	−0.53	−0.63 to −0.41	< 0.001
Illness-related stigma → DLQI score	0.44	0.35 to 0.54	< 0.001
Hope → DLQI score	−0.30	−0.39 to −0.20	< 0.001
Perceived social support → DLQI score	−0.13	−0.22 to −0.04	0.007

Bias-corrected 95% confidence intervals (BC 95% CIs) and two-tailed *P*-values were obtained using bootstrap resampling with 5,000 resamples.

**TABLE 5:** Indirect and total effects in the final structural equation model: standardized estimates based on bias-corrected bootstrapping.

Path	Standardized β	BC 95% CI	Two-tailed P
Specific indirect effects on Hope			
Affected BSA (%) → Illness-related stigma → Hope	−0.16	−0.22 to −0.11	< 0.001
Itch severity → Illness-related stigma → Hope	−0.11	−0.16 to −0.06	< 0.001
Skin pain severity → Illness-related stigma → Hope	−0.11	−0.18 to −0.05	< 0.001
Specific indirect effects on DLQI score			
Illness-related stigma → Hope → DLQI score	0.16	0.11 to 0.22	< 0.001
Affected BSA (%) → Illness-related stigma → DLQI score	0.18	0.13 to 0.24	< 0.001
Itch severity → Illness-related stigma → DLQI score	0.12	0.07 to 0.18	< 0.001
Skin pain severity → Illness-related stigma → DLQI score	0.12	0.06 to 0.19	< 0.001
Affected BSA (%) → Illness-related stigma → Hope → DLQI score	0.06	0.02 to 0.09	< 0.001
Itch severity → Illness-related stigma → Hope → DLQI score	0.04	0.01 to 0.07	< 0.001
Skin pain severity → Illness-related stigma → Hope → DLQI score	0.04	0.01 to 0.07	< 0.001
Total effects			
Illness-related stigma → Hope	−0.53	−0.63 to −0.41	< 0.001
Affected BSA (%) → Hope	−0.16	−0.22 to −0.11	< 0.001
Itch severity → Hope	−0.11	−0.16 to −0.06	< 0.001
Skin pain severity → Hope	−0.11	−0.18 to −0.05	< 0.001
Illness-related stigma → DLQI score	0.60	0.54 to 0.66	< 0.001
Hope → DLQI score	−0.30	−0.39 to −0.20	< 0.001
Perceived social support → DLQI score	−0.13	−0.22 to −0.04	0.007
Affected BSA (%) → DLQI score	0.18	0.13 to 0.24	< 0.001
Itch severity → DLQI score	0.12	0.07 to 0.18	< 0.001
Skin pain severity → DLQI score	0.12	0.06 to 0.19	< 0.001

Indirect and total effects were estimated using bias-corrected bootstrap resampling with 5,000 resamples. Two-tailed *P*-values and BC 95% CIs are reported. In AMOS bootstrap output, standard errors for indirect effects are not routinely provided; consistent with common reporting practice, they are not presented here.

In an exploratory supplementary analysis, Mann–Whitney U tests were used to compare illness-related stigma and DLQI scores according to visible site involvement and high-impact site involvement.

Missing data were examined prior to analysis. The proportion of missing values was minimal (<1%), and missing data were handled using full information maximum likelihood estimation within the SEM framework. The sample size was determined pragmatically based on consecutive recruitment during the prespecified study period, and no formal stopping rules were applied. The final sample size was considered adequate for SEM according to conventional recommendations for model complexity.

Model fit was evaluated using multiple indices: χ^2^/df, comparative fit index (CFI), Tucker–Lewis index (TLI), root mean square error of approximation (RMSEA with 90% CI), and standardized root mean square residual (SRMR). Model fit was considered acceptable based on conventional thresholds (χ^2^/df ≤ 3, CFI/TLI ≥ 0.90, RMSEA ≤ 0.08, SRMR ≤ 0.08).

## 3 Results

### 3.1 Participant characteristics

Demographic and clinical characteristics of the participants are summarized in Table 1.

### 3.2 Descriptive statistics and bivariate correlations

Descriptive statistics of the study variables are presented in Table 2. The study variables showed some variability in their distributional characteristics. Spearman’s rank-order correlation coefficients among the study variables are presented in Table 3. Affected BSA, itch severity, and skin pain severity were positively correlated with illness-related stigma. Illness-related stigma was positively correlated with DLQI scores and negatively correlated with hope. Correlations between perceived social support and stigma or hope were close to zero.

In an exploratory supplementary analysis, patients with visible site involvement had significantly higher illness-related stigma scores and greater DLQI impairment than those without visible site involvement [SSCI: 15.0 (10.0, 23.5) vs. 11.0 (8.0, 17.0), *P* < 0.001; DLQI: 20.0 (13.0, 25.0) vs. 17.0 (12.0, 23.0), *P* = 0.041]. Patients with high-impact site involvement also had significantly higher stigma scores [16.0 (10.0, 24.0) vs. 12.0 (9.0, 18.0), *P* = 0.003], whereas the difference in DLQI was not statistically significant [20.0 (13.0, 25.0) vs. 19.0 (13.0, 24.0), *P* = 0.733].

### 3.3 Measurement model

The measurement component demonstrated acceptable measurement properties (Supplementary Table S1). All standardized factor loadings were statistically significant (*P* < 0.001) and ranged from 0.593 to 0.972. For model identification, one indicator per latent construct was fixed to unity.

For hope, standardized loadings ranged from 0.873 to 0.891, indicating good representation of the latent construct. For perceived social support, factor loadings were 0.794 for family support, 0.593 for friend support, and 0.972 for support from significant others. Although the loading for friend support was comparatively lower, it exceeded the threshold of 0.50 and remained statistically significant.

Convergent validity was supported for both latent constructs, with composite reliability (CR) exceeding 0.70 and average variance extracted (AVE) exceeding 0.50 (Supplementary Table S1), indicating satisfactory construct reliability.

### 3.4 Structural model

**FIGURE 1:**
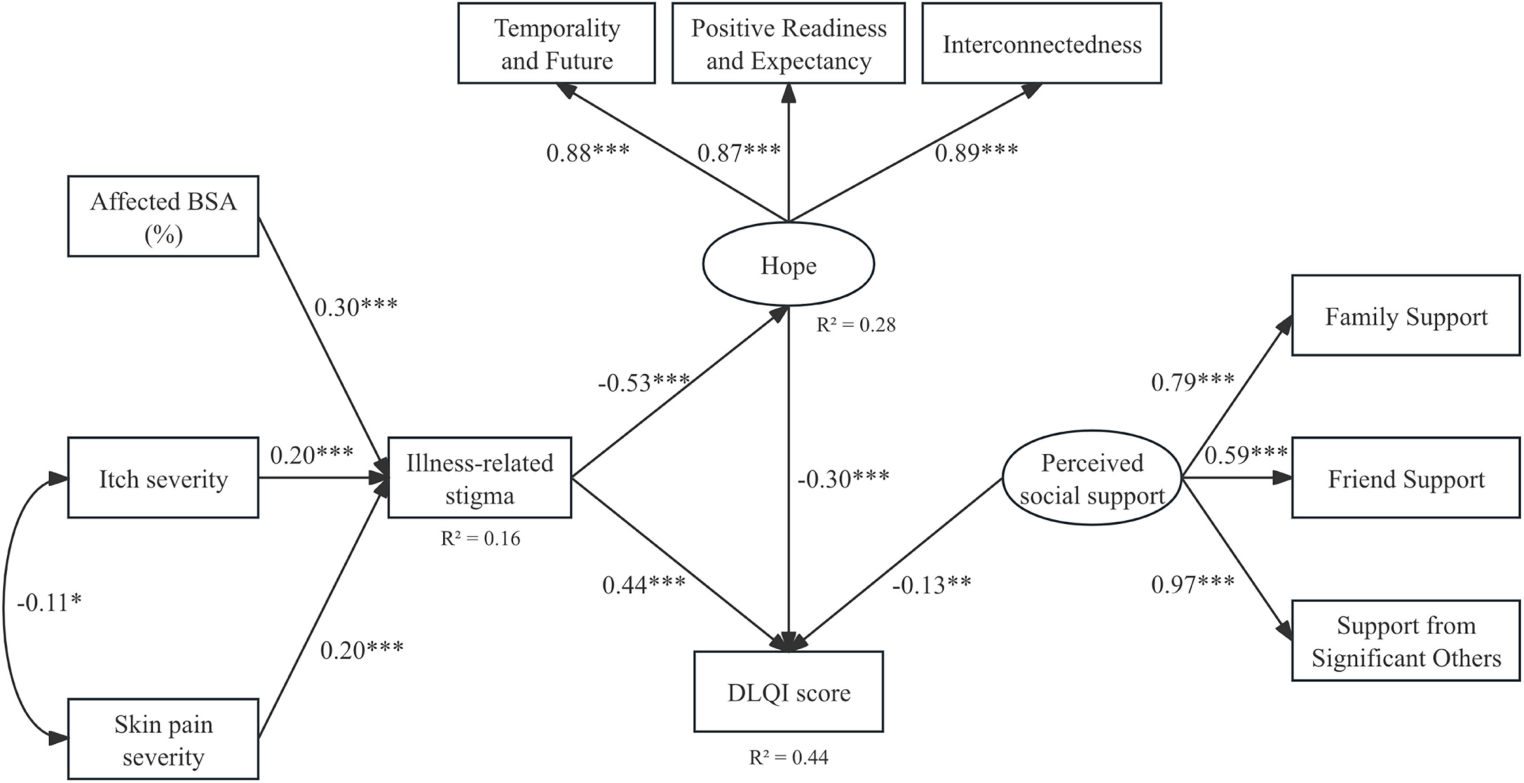
Final structural equation model linking disease severity, illness-related stigma, hope and dermatology-specific quality of life. Standardized path coefficients (β) are shown. *R*^2^-values are shown for endogenous variables. Curved double-headed arrows represent residual covariances. *P < 0.05; **P < 0.01; ***P < 0.001.

The final structural model demonstrated acceptable fit to the data: χ^2^(41) = 83.6, *P* < 0.001; χ^2^/df = 2.04; CFI = 0.977; TLI = 0.969; RMSEA = 0.051 (90% CI 0.036–0.066); and SRMR = 0.058 (Figure 1).

Standardized direct effects are presented in Table 4 and illustrated in Figure 1. Affected BSA (β = 0.30, P < 0.001), itch severity (β = 0.20, P < 0.001), and skin pain severity (β = 0.20, *P* < 0.001) were positively associated with illness-related stigma. Illness-related stigma was negatively associated with hope (β = −0.53, *P* < 0.001) and positively associated with DLQI scores (β = 0.44, *P* < 0.001). Hope was independently associated with lower DLQI impairment (β = −0.30, *P* < 0.001). Perceived social support was directly associated with lower DLQI scores (β = −0.13, *P* = 0.007). The model accounted for 16% of the variance in illness-related stigma, 28% in hope, and 44% in DLQI score.

Among the covariances specified between exogenous variables, only the residual covariance between itch severity and skin pain severity was retained (β = −0.11, *P* < 0.05); all other tested covariances were non-significant and constrained to zero.

### 3.5 Indirect and total effects

Bias-corrected bootstrapped indirect and total effects are reported in Table 5. The three disease severity indicators showed significant indirect effects on hope via illness-related stigma, and on DLQI score through illness-related stigma and the serial pathway from stigma to hope (all BC 95% CIs excluded zero). Illness-related stigma also demonstrated a significant indirect effect on DLQI score via hope. Corresponding total effects were statistically significant (all BC 95% CIs excluded zero).

The hypothesized path from illness-related stigma to perceived social support was non-significant and excluded from the final model; consequently, no indirect effects via perceived social support were observed.

## 4 Discussion

In this inpatient sample, greater objective and subjective disease severity was associated with higher levels of illness-related stigma, which was further related to poorer dermatology-specific HRQoL and lower hope. These findings are consistent with multinational data demonstrating that perceived stigmatization is highly prevalent across dermatological conditions and constitutes a substantial component of psychosocial burden (5). Beyond empirical observations, stigma has been conceptualized as a social–cognitive process involving labeling, stereotyping and status loss, which may influence self-perception and coping responses (6). Within stress–appraisal frameworks of chronic illness, disease-related stressors may influence psychological outcomes through cognitive appraisal processes that shape the perceived meaning and controllability of disease-related stressors (19). Our findings support considering stigma as a proximal psychological process that may link clinical manifestations and dermatology-specific HRQoL impairment.

The present findings further suggest that symptom-related features of inflammatory dermatoses—particularly greater lesion extent (BSA), pruritus and discomfort—may be associated with heightened perceived social evaluation and internalized stigma. Inflammatory skin diseases often involve fluctuating lesions and persistent symptoms that may increase patients’ awareness of social scrutiny. Empirical evidence indicates that both objective severity indicators and subjective symptom intensity are associated with greater perceived stigmatization in dermatological populations (5). Data from the EADV “burden of skin diseases” project further show that embarrassment and perceived rejection are common experiences among European patients with skin disease (20). Recent evidence from Asia also supports substantial psychosocial and quality-of-life burden in dermatological populations. A systematic review of atopic dermatitis in Asian countries identified embarrassment as a prominent dimension of HRQoL impairment, while recent studies from China have shown marked DLQI impairment in psoriasis and highlighted the contribution of lesion location to HRQoL burden (21–23). Notably, symptom-related distress, particularly persistent itch, has been associated with greater stress and experiences of stigmatization, suggesting that subjective symptom burden may contribute to stigma internalization beyond visible disease severity (24). In an exploratory supplementary analysis, involvement of visible body sites was also associated with higher stigma and greater dermatology-specific HRQoL impairment, whereas high-impact site involvement was associated with higher stigma only. These findings suggest that lesion location, in addition to overall disease extent, may contribute to psychosocial burden in patients with chronic inflammatory skin diseases. Together, these findings support conceptualizing stigma as a psychological response to disease burden rather than merely a correlate of poorer HRQoL.

Illness-related stigma may play an important role in dermatology-specific HRQoL because it represents an internalized, self-referential stressor. Stigma processes extend beyond external social reactions and involve anticipated rejection, concealment or avoidance, and diminished self-worth, which may shape illness interpretation and expectations of social participation (6). Contemporary health-related stigma frameworks emphasize internalized and anticipated stigma as key mechanisms through which chronic health conditions generate psychological burden (25). Within stress–appraisal models, such internalization may be associated with diminished adaptive coping and reduced future-oriented cognitive appraisals, potentially contributing to lower levels of hope. Hope theory conceptualizes hope as a cognitive–motivational resource characterized by goal-directed thinking and perceived pathways toward desired outcomes (26). Across chronic illness populations, higher levels of hope have been associated with better quality of life, lower psychological distress, and better adjustment, supporting its role as a psychological resource rather than merely reflecting symptom severity (27, 28).

Accordingly, the present findings are consistent with the proposed pathway model in which higher levels of stigma were associated with lower levels of hope, which in turn related to poorer dermatology-specific HRQoL. This interpretation aligns with contemporary stigma frameworks suggesting that internalized stigma may erode psychological resources relevant to adaptive coping and thereby contribute to adverse patient-reported outcomes (25, 26).

Perceived social support did not emerge as a significant mediator in the present model, but remained independently associated with dermatology-specific HRQoL. This pattern is conceptually consistent with the buffering hypothesis, which proposes that social support may attenuate the association between stressors and health outcomes without necessarily operating as a proximal psychological mechanism (29). In dermatological populations, perceived support has been shown to vary across domains (family, friends and significant others), with friend-related support often reported as comparatively lower (30). Empirical evidence further suggests that perceived support may be reduced in patients with chronic skin disease compared with healthy controls and may be associated with disease visibility and symptom burden (9).

In the present structural model, social support may contribute to patient-reported outcomes through broader contextual buffering effects rather than through the internal stigma–hope pathway identified in the model. These findings underscore the importance of integrating proximal cognitive processes with broader interpersonal resources when examining psychosocial influences on HRQoL.

An additional observation was a modest but statistically significant negative residual covariance between itch severity and skin pain severity. Importantly, this covariance reflects shared variance remaining after accounting for the specified structural paths rather than a simple bivariate association. Neurophysiological and experimental research suggests that itch and pain are closely related yet partially antagonistic sensory modalities (31, 32). Experimental findings indicate that nociceptive input can suppress itch processing through central inhibitory mechanisms (31). In clinical contexts, patients frequently describe scratching as providing transient “painful relief,” consistent with counter-irritation phenomena (33). Although the present cross-sectional design does not permit mechanistic inference, the observed negative residual covariance may be compatible with the possibility of modality-specific regulatory interactions between itch and pain rather than reflecting simple collinearity between symptom measures.

These findings have practical implications for the inpatient management of chronic inflammatory skin diseases. Beyond controlling cutaneous inflammation, routine care may consider incorporating brief screening for illness-related stigma, given its association with poorer dermatology-specific HRQoL. Multicenter dermatology data from outpatient populations indicate that perceived stigmatization is common and clinically meaningful across skin conditions, underscoring the relevance of systematic identification (5).

The independent association between hope and dermatology-specific HRQoL further suggests that psychological resources may represent relevant components of comprehensive care. Although psychodermatological interventions are variably implemented in routine practice, a meta-analysis indicates that psychological interventions targeting coping and emotional adjustment can improve patient-reported outcomes in selected dermatological populations (34).

Finally, the direct association between perceived social support and HRQoL highlights the potential value of strengthening practical and emotional support structures. Collectively, these findings suggest the potential value of integrating routine psychosocial assessment and screening, alongside supportive strategies, into standard dermatologic care.

The final model was derived from a prespecified theory-driven baseline model and refined by removing non-significant direct paths and unsupported associations while retaining theoretically plausible relationships. Together with the supplementary baseline model diagram (Supplementary Figure S1), this refinement process supports a parsimonious representation of the data in which stigma and hope emerged as the principal proximal pathways linking disease burden to dermatology-specific HRQoL.

This study has several strengths. The large inpatient sample (N = 404) provided a robust dataset for structural equation modeling with bootstrapped estimation. The integration of objective clinical indicators, subjective symptom severity, and psychosocial constructs within a unified model enabled the simultaneous examination of direct and indirect associations while accounting for latent measurement structures. Clear specification of model structure and parameter estimation enhances transparency and interpretability.

Several limitations exist. First, the cross-sectional design precludes causal inference, and longitudinal studies are required to clarify temporal relationships among disease severity, stigma, psychological resources, and HRQoL. Second, the single-center inpatient sample limits generalizability to outpatient or community settings. Third, reliance on self-reported psychosocial measures introduces potential common method bias; while SEM accounts for measurement error in latent constructs, some shared variance may still exist. Finally, grouping heterogeneous chronic inflammatory skin diseases enhances ecological validity but may obscure condition-specific patterns, which require further investigation. In addition, although an exploratory supplementary analysis suggested that lesion location may be relevant to stigma and dermatology-specific HRQoL, site involvement was not incorporated into the prespecified structural equation model and was not quantified in terms of site-specific severity. Future studies should further examine the role of lesion visibility and high-impact body sites alongside overall disease extent.

## 5 Conclusion

In conclusion, the present study supports a pattern of associations in which clinical disease burden is related to poorer dermatology-specific HRQoL beyond physical severity alone, partly through illness-related stigma and reduced hope. By identifying stigma as a proximal psychological correlate and hope as a clinically relevant psychological resource, these findings highlight the potential value of integrating psychosocial assessment into routine dermatologic care. Future longitudinal and multicenter studies are needed to clarify temporal relationships and to evaluate interventions targeting stigma-related burden and psychological resources in patients with chronic inflammatory skin diseases.

## Data Availability

The raw data supporting the conclusions of this article will be made available by the authors, without undue reservation.
