# Supplementary material for: Illness-related stigma mediates the association between disease severity and dermatology- specific quality of life in chronic inflammatory skin diseases: a structural equation modeling study
Source: Front Med (Lausanne). 2026 May 7;13:1821716. doi: 10.3389/fmed.2026.1821716 (PMC13190396; doi:10.3389/fmed.2026.1821716)
Supplement: Supplementary file 1 [file Table_1.docx]

Supplementary Material

# Supplementary Data

To assess the robustness of the hypothesized mediation model, a supplementary sensitivity analysis was performed by adding sex, age, education level, and financial burden as covariates predicting DLQI score. The structural and measurement components were otherwise specified identically to the final model reported in the main text.

As in the primary model, covariances among the severity indicators were initially estimated. Only the covariance between itch severity and skin pain severity was retained in the final sensitivity model, whereas the covariances between affected BSA and itch or skin pain were non-significant and were therefore constrained to zero for parsimony.

All other exogenous variables in the sensitivity model were freely allowed to covary.

# Supplementary Figures

**Figure S1. Prespecified baseline structural equation model.**


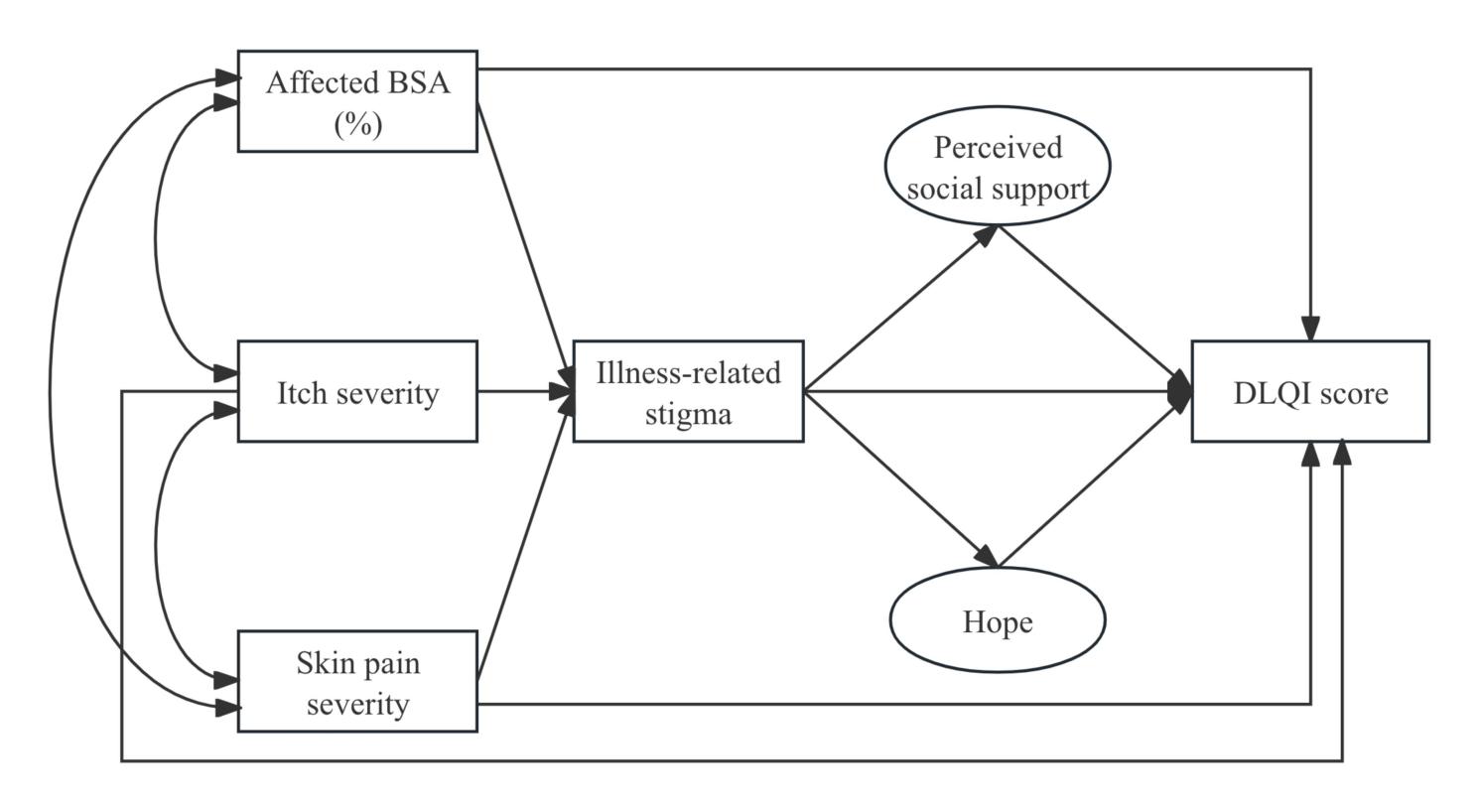
The diagram illustrates the hypothesised relationships among disease severity indicators, illness-related stigma, hope, perceived social support, and dermatology-specific quality of life before model refinement. Covariances among exogenous severity indicators were freely estimated in the baseline model. No parameter estimates are shown.

# Supplementary Tables

**Table S1. Standardized factor loadings for the measurement model.**

| Indicator | Latent variable | Standardized factor loading | SE | C.R. | P |
| --- | --- | --- | --- | --- | --- |
| Temporality and Future | Hope | 0.88 | 0.04 | 24.08 | <0.001 |
| Positive Readiness and Expectancy | Hope | 0.87 | 0.05 | 23.37 | <0.001 |
| Interconnectedness^a^ | Hope | 0.89 | — | — | — |
| Family Support | Perceived social support | 0.79 | 0.05 | 15.12 | <0.001 |
| Friend Support | Perceived social support | 0.59 | 0.06 | 11.68 | <0.001 |
| Support from Significant Others^a^ | Perceived social support | 0.97 | — | — | — |
| ^a^ Reference indicator fixed for identification. Standardized factor loadings estimated by maximum likelihood are presented. C.R., critical ratio; CR, composite reliability; AVE, average variance extracted. Convergent validity was supported for both latent constructs: hope (CR = 0.91, AVE = 0.78) and perceived social support (CR = 0.84, AVE = 0.64). | | | | | |

**Table S2A. Model fit indices: main model versus sensitivity model.**

| Fit index | Main model (final structural model) | Sensitivity model (with added covariates*) |
| --- | --- | --- |
| χ² (df) | 83.6 (41) | 204.30 (81) |
| χ²/df | 2.04 | 2.52 |
| CFI | 0.977 | 0.939 |
| TLI | 0.969 | 0.921 |
| RMSEA (90% CI) | 0.051 (0.036–0.066) | 0.061 (0.051–0.072) |
| SRMR | 0.058 | 0.0645 |
| * Covariates included sex, age, education level, and financial burden, specified as predictors of DLQI score. All other exogenous variables were freely allowed to covary. | | |

**Table S2B. Standardized structural path coefficients: main model versus sensitivity model.**

| Path | Main model (β) | Sensitivity model (β) |
| --- | --- | --- |
| Affected BSA (%) → Illness-related stigma | 0.299 | 0.299 |
| Itch severity → Illness-related stigma | 0.202 | 0.202 |
| Skin pain severity → Illness-related stigma | 0.203 | 0.203 |
| Illness-related stigma → Hope | −0.527 | −0.527 |
| Illness-related stigma → DLQI score | 0.444 | 0.441 |
| Hope → DLQI score | −0.298 | −0.299 |
| Perceived social support → DLQI score | −0.128 | −0.128 |
| Standardized path coefficients (β) are presented. Differences between the two models were minimal (< 0.005 in absolute value), indicating that the main structural associations were robust to covariate adjustment. | | |
